# A two-mediator serial mediation chain of the association between social isolation and impaired sleep in old age

**DOI:** 10.1038/s41598-022-26840-5

**Published:** 2022-12-28

**Authors:** Razak M. Gyasi, Kabila Abass, Alexander Yao Segbefia, Kwadwo Afriyie, Edward Asamoah, Mary Sefa Boampong, Anokye M. Adam, Ellis Owusu-Dabo

**Affiliations:** 1 Aging and Development Program, African Population and Health Research Center (APHRC), Manga Close, Off-Kirawa Road, P. O. Box 10787-00100, Nairobi, Kenya; 2 National Centre for Naturopathic Medicine, Faculty of Health, Southern Cross University, Lismore, NSW Australia; 3 Department of Geography and Rural Development, Kwame Nkrumah University of Science and Technology, Kumasi, Ghana; 4 Department of Sociology and Social Work, Kwame Nkrumah University of Science and Technology, Kumasi, Ghana; 5 Department of Finance, School of Business, University of Cape Coast, Cape Coast, Ghana; 6 School of Public Health, Kwame Nkrumah University of Science and Technology, Kumasi, Ghana

**Keywords:** Psychology, Medical research, Risk factors

## Abstract

Poor sleep is a long-term public health issue that has become increasingly prevalent among socially isolated older adults. However, research on the mechanisms explaining the link between social isolation and impaired sleep (IS) remains limited, particularly in low- and middle-income countries. This study explored the serial mediating effects of loneliness and mental distress on the association of social isolation with IS among Ghanaian older adults. We analyzed data from 1201 adults aged ≥ 50 from Ghana's AgeHeaPsyWel–HeaSeeB study (mean age = 66.14, *SD* = 11.85, age range = 50–111; women = 63.28%). Measures included the UCLA 3-item Loneliness Scale, modified Berkman–Syme Social Network Index, Sleep Quality Scale, and Mental Distress Questionnaire. We used bootstrapping techniques from Hayes’ PROCESS macro program to estimate the hypothesized serial mediation. Social isolation was significantly associated with IS (β = 0.242, *p* < 0.001). Crucially, social isolation indirectly predicted IS via three significant mediating pathways. Loneliness accounted for 17.6% (β = 0.054, CI = 0.096, 0.016), mental distress accounted for 6.5% (β = 0.020, 95% CI = 0.004, 0.040), and loneliness and mental distress accounted for 32.2% (β = 0.099, 95% CI = 0.065, 0.138) of the overall effect. The total mediating effect was 56.4%. These findings suggest that the social isolation-sleep link is respectively and serially explained by loneliness and mental distress. Social integrative interventions for sleep quality in old age should target mental and emotional well-being.

## Introduction

Sleep is a fundamental bio-physiological and psychological process for human health. Sufficient sleep duration and sleep quality regulate and maintain well-being^1–3^, particularly in later life. However, poor sleep remains prevalent in old age^1^. Recent estimates indicate that 22–53% of older people experience sleep disorders^4^. Several population-based studies with older samples have shown associations between sleep disturbance and clinical events, including cardiovascular risk factors and diseases^5,6^, neurodegenerative conditions such as Parkinson’s disease^7^ and stroke^8^, reduced quality of life^9^, and increased risk of premature mortality^10,11^. Therefore, a better understanding of the risk factors of poor sleep may inform decisions to improve well-being and performance for healthy aging.

Theorized as the objectively measured deficit of social contact with relevant others, social isolation is linked with emotional distress and cognitive deterioration, including impaired sleep (IS)^12^. It is distinct from loneliness, emotional distress experience, and the perception of unsatisfying social relationships^13^. The socially isolated are at a higher risk of sleep disturbances than those in a denser, more robust constellation of social networks^14^. Cross-sectional and longitudinal analyses among 140,423 adults found that socially isolated were more likely to experience insufficient sleep^15^. A recent systematic review involving 11 studies showed that social isolation is plausibly associated with IS^16^. Using the IN-DEPTH WHO-SAGE data, Stranges et al.^17^ found that not living in partnership was related to a higher prevalence of sleep problems. The precise mechanisms underlying these geriatric syndromes remain unclear despite the apparent association of social isolation with suboptimal sleep. However, theoretically, several hypotheses have been proposed through which social isolation may promote poor sleep and IS. For example, social isolation may increase the perception of poor belonging and social connectedness, emotional grief, and fatigue which, in turn, reduce the required energy for uninterrupted sleep^18,19^. Moreover, the risk of loneliness and cognitive dysfunction sequelae which are attendant to poor social attachment may lead to intense unhealthy sleep among older people^19–21^.

The loneliness- and mental dysfunction-specific roles in the association of social isolation with sleep may be due to an exacerbation and the rise in transient and chronic perceived poor belonging and alteration in the inflammatory biomarkers that may inhibit sleep quality^22^. Prior studies established social isolation as a risk factor for feeling lonely and having mental distress^23–25^. Published literature has linked loneliness to mental health challenges among older people^26,27^. For example, a systematic review and meta-analysis of 27 studies found that loneliness correlated with self-reported sleep disturbance (*r* = 0.28, 95% CI = 0.24–0.33)^21^. In addition, two systematic reviews of nine and 51 studies each concluded that mental health problems such as depression and anxiety increase sleep disturbance^28,29^. In turn, loneliness and mental distress have been shown in multiple cross-sectional and longitudinal studies to be associated with an increased risk of poor sleep, although the relationship could be bidirectional^30–32^. Thus, there is a possibility that poor sleep quality may lead to loneliness and social isolation, particularly in later life. For example, Ben Simon et al.^33^ found that poor sleep can trigger loneliness and social withdrawal. Many of these studies have adjusted for social isolation to estimate independent associations between loneliness/mental distress and IS. However, no previous research has used principled analytic techniques to quantify how much social isolation risks for IS are mediated via loneliness and mental distress. Understanding the potential mediating effects of loneliness and mental distress on the relationship between social isolation and sleep may be crucial in tackling the rising burden of sleep-related disorders.

**Figure 1:**
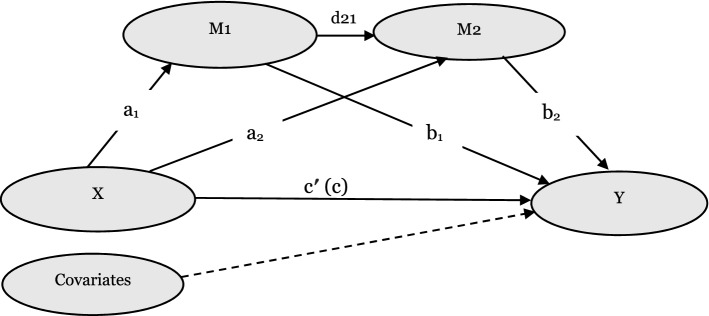
A hypothesized serial multiple mediation model. X is the predictor, M1 and M2 are mediators, and Y is the outcome variable. a_1_, a_2_, b_1_, b_2_, c, c′, and d_21_ represent path coefficients.

The current study aimed to explore the direct and indirect associations between social isolation and IS among older adults in Ghana using serial and cascading mediating estimations. The theoretical hypotheses in Fig. 1 are proposed to guide the study. Specifically,

### Hypothesis 1

There is a statistically significant positive association between social isolation and IS in old age (total effect, c);

### Hypothesis 2

There is a specific indirect effect of social isolation on IS through loneliness (indirect effect a_1_b_1_);

### Hypothesis 3

There is a specific indirect effect of social isolation on IS through mental distress (indirect effect a_2_b_2_);

### Hypothesis 4

There is a serial multiple mediation effect of social isolation on IS through loneliness and mental distress (cascading indirect effect a_1_d_21_b_2_).

## Methods

This study utilized data from the AgeHeaPsyWel–HeaSeeB Study focusing on the health and health-seeking-behavior dynamics of community-dwelling adults aged ≥ 50 years^34^. The study was conducted in the Ashanti Region of Ghana and included six rural/urban districts. The study devised a multi-stage stratified cluster sampling procedure. The sample size was estimated with the WHO’s estimation formula , (${\uppi }$: expected prevalence; : design effect)^35^. We assumed a 5% margin of error, 95% confidence interval, 1.5 design effect, 5% type 1 error, 15% type 2 error, and 50% conservative prevalence. The proportion of adults ≥ 50 years in the study region was unknown ${\uppi } = 50{\text{\% }} = 0.5$ and $\mho_{{_{{{\upalpha }/2}} }} = 1.96$. These parameters yielded 901 as the minimum required sample size. Considering the loss and refusal to participate and ensuring the generalizability of findings, we oversampled by 38%, attaining a sample of 1247. This achieved a statistical power of 85% at a 5% (two-sided) significance level to detect an odds ratio of ≥ 2.

**Figure 2:**
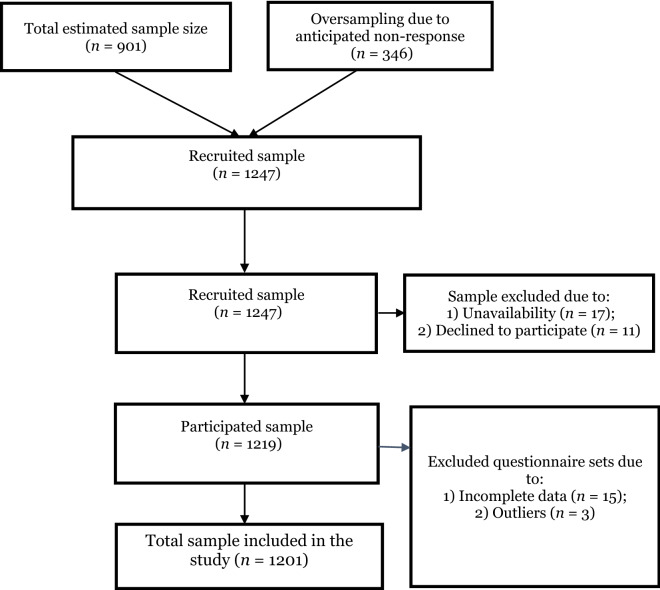
Flow chart of the selection of study participants.

Participants were included in the study if they were available and willing to participate. However, approximately 3.7% were subsequently excluded based on various reasons. Thus, 17 participants (1.4%) were not available during data collection, 11 participants (0.9%) declined to participate, 15 participants (1.2%) missing essential data, and 3 participants (0.2%) contained outliers, leaving the final analytic sample of 1201 with a response rate of 96.31% (Fig. 2). Participants were approached in their homes and informed of the purpose/procedure of the research upon their recruitment. They completed written informed consent and questionnaires through trained interviewers. The study procedure was reviewed and approved by the CHRPE, School of Medical Sciences, Kwame Nkrumah University of Science and Technology, and Komfo Anokye Teaching Hospital, Kumasi, Ghana (Ref: CHRPE/AP/507/16). Moreover, all methods and procedures involved in this study were firmly performed in accordance with the relevant guidelines and regulations.

### Ethical approval and informed consent

The study protocol received ethics approval from the Committee on Human Research Publication and Ethics, School of Medical Sciences, Kwame Nkrumah University of Science and Technology, and Komfo Anokye Teaching Hospital, Kumasi, Ghana (Ref: CHRPE/AP/507/16). Written informed consent was obtained from all individual participants included in the study.

## Measures

### Impaired sleep (IS)

IS was assessed using a fatigue-related questionnaire with modest-to-high sensitivity for detecting clinically relevant obstructive sleep quality in the general population^36^. Participants were asked: “Overall, in the last 30 days, how much of a problem did you have with sleeping, e.g., falling asleep, waking up frequently during the night, or waking up too early in the morning?” and “Overall in the last 30 days, how much of a problem did you have due to not feeling rested and refreshed during the day (for example, feeling tired, not having energy)?” Each item had 5-point Likert response options: none = 1, mild = 2, moderate = 3, severe = 4, extreme = 5. These items have been used in previous SSA studies^37^. A latent sleep quality score was generated with an increasing score indicating higher levels of IS ($\propto$= 0.830).

### Social isolation

Social isolation was assessed using an adapted Berkman–Syme Social Network Index^38^. We included six (6) domains as indicators for social isolation: (1) marriage, (2) contact with friends and relatives, (3) social participation, (4) availability of someone to take you to the hospital, (5) availability of someone to share concerns, and (6) feeling a strong emotional bond with others. We assigned a score of 1 for never married/widowed/separated/divorced, and 0 for otherwise. For family/friends contact and social participation, we set 1 point each for never/once/twice per year, and 0 for once/twice per month/once/twice per week/almost every day. For the availability of someone to take you to the hospital, someone to share concerns, and feeling a strong emotional bond with others, we assigned 1 point each for completely false/somewhat false/neutral, and 0 for partially true/completely true. We created social isolation index (range: 0–6); higher scores indicate higher levels of social isolation ($\propto$= 0.891).

### Loneliness

Loneliness was assessed using the validated 3-item UCLA Loneliness Scale^39^. The 3-item UCLA Loneliness Scale has a high concurrent and discriminant validity and good consistency^39^ with Cronbach’s alpha, $\propto$ = 0.819 in the present study. The 3-item UCLA Loneliness Scale includes three questions asking the frequency participants feel they lack companionship, are left out, or are isolated from others rated on a 3-point Likert-type scale: “Hardly ever or never” (1 point), “Some of the time” (2 points), or “Often” (3 points). A total score (range: 3–9) was used to measure loneliness, with higher scores indicating greater levels of loneliness.

### Mental distress

Mental distress was assessed as a composite score computed by summing up six indicators of the mental/psychological functioning of the respondents^40^. The items were evaluated with widely used self-rated and cross-culturally validated items: “Over the past four weeks, have you been (1) happy, (2) sad/depressed, (3) nervous/uneasy, (4) restless/fidgety/bored, (5) hopeless, and (6) worthless/having no value” with four-point Likert-like options: 1 = none of the time, 2 = little of the time, 3 = most of the time, 4 = all of the time. “Happy” was reverse-coded to ensure comparability. The overall score ranged from 6 to 24, with higher scores indicating higher levels of mental distress ($\propto$= 0.897).

### Covariates

Informed by previous research^16,17,22^, we selected and controlled for potential confounders in this analysis. Age (years), sex (male/female), residential type (rural/urban), education (basic/never, high school, tertiary), and income (Cedis) were included. Health behaviors included alcohol intake (no, yes) and physical activity (PA), assessed using the International Physical Activity Questionnaire (IPAQ-SF), measuring the dimensions of PA intensity over the past seven days^41^. The responses ranged from 0 to 7, with higher scores indicating higher levels of PA. Regarding health variables, self-rated health was assessed with a single item asking the participants to rate their current health: excellent = 1, poor = 5, with higher scores reflecting poor health status.

Physical function impairment was assessed with seven functional impairments and mobility-related deficiencies in older persons^42^. Each item was scored on a four-point Likert-like option: not limited at all = 1, much limited = 4. The total score ranged from 7 to 28; higher scores on the scale represented more elevated levels of functional impairment ($\propto$= 0.830). Pain interference was assessed with a single item from the bodily pain subscale of the Medical Outcomes Study Short Form-36 (MOS SF-36)^40^ using a 1–5 rating scale with established reliability and validity: The responses included not at all = 1, extreme = 5 with higher scores, suggesting higher levels of pain interference^43^.

### Statistical analysis

Statistical analyses were performed using SPSS 25.0 (SPSS, Inc., IBM, Armonk, NY, USA) with $\propto$=0.05 as the statistically significant level. We employed descriptive statistics to describe the attributes of the participants. Categorical variables were presented as counts/proportions, while continuous variables were depicted as means/standard deviations. Next, zero-order correlations were computed to examine the association between the core study variables, including IS, social isolation, loneliness, and mental distress. The *p* values were adjusted for multiple correlations by a Bonferroni correction to avoid the risk of a type I error.

To evaluate the hypotheses and to understand the potential mechanistic pathways linking social isolation and IS, we conducted bootstrapping analyses with social isolation as the predictor, loneliness and mental distress as the mediators, and IS as the outcome measure using the SPSS PROCESS macro version 4.0 software^44^. Serial multiple mediation analyses (Model 6) of the PROCESS macro were based on bootstrapping using 95% confidence intervals (CI). Bootstrapping for indirect effects was set at 5000 samples. If the 95% CI of the mediation effect did not include zero (0), the mediation effect was statistically significant at the 0.05 threshold. The macro allowed for calculating and testing direct, total, and indirect effects. Bias-corrected bootstrapped CIs were used to assess the statistical significance of the indirect effects. The mediation models included age (in years), sex (male = 1, female = 2), residential setting (rural = 1, urban = 2), education (never/primary = 1, secondary = 2, tertiary = 3), income levels (in Ghana Cedis), alcohol consumption (no = 1, yes = 2), PA participation (continuous), self-rated health (continuous), pain interference (continuous), number of chronic condition (continuous, from a list of chronic physical conditions, including hypertension, diabetes, stroke, kidney diseases, asthma, cancers, lung diseases, and arthritis), and functional limitations (continuous) as confounding variables.

## Results

### Descriptive statistics and correlation analysis

The total analytic sample comprised 1201 participants. The mean age was 66.14(± 11.85) and 63.28% were women. The majority lived in urban areas (55.04%), attained primary school level/never (86.18%), and were non-alcohol consumers (68.50%). The mean income was 307.98 Cedis. On average, participants reported 3.32 multimorbidity, and self-rated health was 3.01. The mean number of impaired functionalities was 8.91 (Table 1). The average score of IS (4.89 ± 1.89), social isolation (10.79 ± 1.45), loneliness (5.28 ± 2.45), and mental distress (11.17 ± 4.28) were revealed (Table 2). Pearson’s zero-order correlation matrix for the core variables is shown in Table 2. Social isolation (*r* = 0.236, *p* < 0.001), loneliness (*r* = 0.173, *p* < 0.001), and mental distress (*r* = 0.275, *p* < 0.001) were significantly and positively interrelated with IS. Loneliness correlated strongly with mental distress (*r* = 0.782, *p* < 0.001). Social isolation correlated with loneliness (*r* = 0.302, *p* < 0.001) and mental distress (*r* = 0.279, *p* < 0.001).

**Table 1:** Control variables and the characteristics of the study participants (*N* = 1201).

Variable	%/M(± SD): Range
Age (in years)	66.14(± 11.85): 50–111
Sex	63.28% Female
	36.72% Male
Setting	44.96% Rural
	55.04% Urban
Income level	307.98 (± 338.79)
Educational level	86.18% None or primary
	8.66% Secondary
	5.16% Tertiary
Alcohol intake	68.50% No
	31.50% Yes
Physical activity	3.01(± 1.47)
Self-rated health	3.44(± 0.84): 1–5
Pain interference	3.03(± 1.26): 1–5
Number of chronic physical Conditions	3.32(± 3.97): 0–5
Functional limitations	8.91(± 2.15): 7–28

*N* valid frequency, *M* mean, *SD* standard deviation.

**Table 2:** Means, standard deviations, and zero-order Pearson’s correlations between core study variables with Bonferroni Correction for multiple comparisons.

Variable	Mean	(± SD)	IS	Social isolation	Loneliness	Mental distress
IS	4.888	(1.889)	1			
Social isolation	10.794	(1.462)	0.236**	1		
Loneliness	1.762	(0.816)	0.173**	0.302**	1	
Mental distress	18.729	(3.689)	0.275**	0.279**	0.782**	1

*IS* impaired sleep.

**p < 0.001.

### A serial mediation modeling

**Figure 3:**
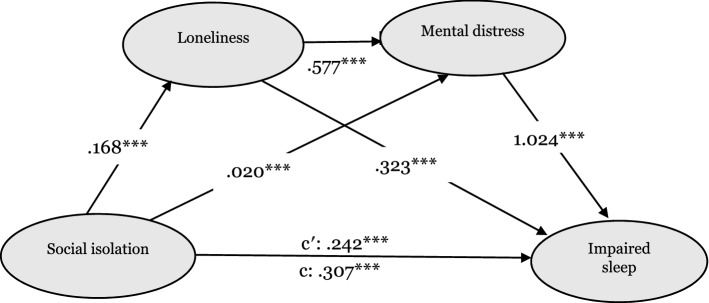
A serial multiple mediation model of the association between social isolation and impaired sleep through loneliness and mental distress. Path unstandardized coefficients are depicted. C′ denotes the direct effect of social isolation on impaired sleep; c denotes the total effect of social isolation on impaired sleep via mediators.

We constructed adjusted multiple mediation models to investigate the mediating effects of loneliness and mental distress in the association of social isolation with IS (Table 3). The analyses revealed that the model's path coefficients (latent variable correlations) were significant. Thus, the paths from social isolation to loneliness (a1: *β* = 0.168, *p* < 0.001), mental distress (a2: *β* = 0.020, *p* < 0.001), and IS (c′: *β* = 0.242, *p* < 0.001) were significant. Loneliness was positively associated with both mental distress (d21: *β* = 0.577, *p* < 0.001), and IS (b1: *β* = 0.323, *p* < 0.001). Mental distress was positively associated with IS (b2: *β* = 1.024, *p* < 0.001). The mediation path model is shown in Fig. 3. The direct path from social isolation to IS remained significant after adding the potential mediators indicating that loneliness and mental distress partially mediated the social isolation-IS link.

**Table 3:** Regression coefficients in the serial multiple mediation analysis (*N* = 1201).

Outcome measure	Predictor variable	*R*	*R*^2^	*F*	β	*t*	95% CI
Loneliness	Social isolation	0.301	0.091	101.01	0.168***	44.79	0.135, 0.200
Mental distress	Social isolation	0.0784	0.614	548.81	0.020*	2.41	0.004, 0.036
	Loneliness	–	–	–	0.577***	31.90	0.542, 0.613
IS	Social isolation	0.334	0.111	42.37	0.242***	6.17	0.165, 0.318
	Loneliness	–	–	–	0.323**	2.74	0.554, 0.091
	Mental distress	–	–	–	1.024***	6.63	0.721, 1.328

Each model was adjusted for age, sex, residential status, education level, income level, marital status, physical activity, self-rated health, multimorbidity, and functional limitations.

*IS* impaired sleep.

***p˂0.001; **p˂0.005; *p˂0.05.

**Figure 4:**
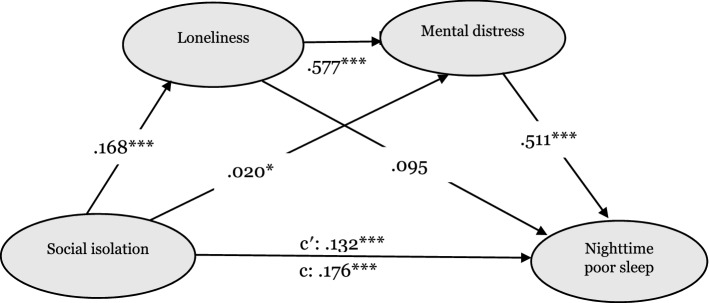
A serial multiple mediation model of the association between social isolation and experiencing nighttime poor sleep (e.g. falling asleep, waking up frequently during the night or waking up too early in the morning) through loneliness and mental distress. Path unstandardized coefficients are depicted. C′ denotes the direct effect of social isolation on impaired sleep; c denotes the total effect of social isolation on impaired sleep via mediators. *p < 0.05, ***p < 0.001.

**Figure 5:**
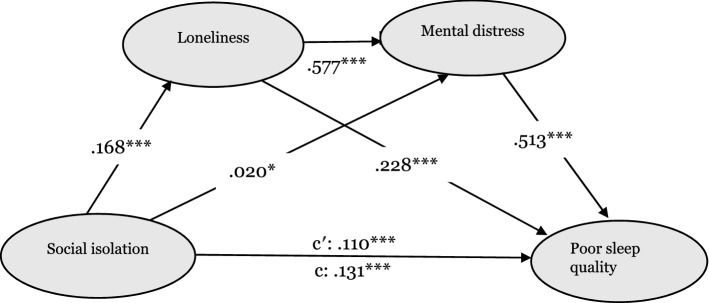
A serial multiple mediation model of the association between social isolation and poor sleep quality (e.g., not feeling rested after waking up from sleep in the morning) through loneliness and mental distress. Path unstandardized coefficients are depicted. C′ denotes the direct effect of social isolation on impaired sleep; c denotes the total effect of social isolation on impaired sleep via mediators. *p < 0.05, ***p < 0.001.

In secondary analyses, we performed stratified IS analyses for nighttime poor sleep (Fig. 4) and poor sleep quality (Fig. 5). In terms of nighttime poor sleep, social isolation significantly and positively influenced loneliness (*β* = 0.168, *p* < 0.001), mental distress (*β* = 0.020, *p* < 0.05), and nighttime poor sleep (*β* = 0.131, *p* < 0.001). Loneliness was positively related to mental distress (*β* = 0.577, *p* < 0.001), but not nighttime poor sleep (*β* = 0.095, *p* > 0.05) while mental distress positively related to nighttime poor sleep (*β* = 0.511, *p* < 0.001). Regarding poor sleep quality, social isolation positively predicted loneliness (*β* = 0.168, *p* < 0.001), mental distress (*β* = 0.020, *p* < 0.05), and poor sleep quality (*β* = 0.110, *p* < 0.001). Loneliness was associated with increased mental distress (*β* = 0.577, *p* < 0.001), and poor sleep quality (*β* = 0.228, *p* > 0.001). Mental distress finally increased poor sleep quality (*β* = 0.513, *p* < 0.001). The paths from social isolation to nighttime poor sleep, as well as poor sleep quality, were significant after including the mediators. This suggests that loneliness and mental distress partially mediated the association of social isolation with nighttime poor sleep and poor sleep quality.

### Bootstrapping test of mediating effects

Table 4 presents the total, direct, and indirect effects. The bootstrap-derived 95% CI estimation procedure with 5000 bootstrap samples did not include zero for any outcomes, suggesting a significant indirect effect of social isolation via loneliness and mental distress on IS net of potential confounders. IS was found to be indirectly affected by social isolation through significant mediation pathways, including loneliness (*β* = 0.054, Boot*SE* = 0.016, 95% CI = 0.096, 0.016) accounting for 17.59% of the total effect, mental distress (*β* = 0.020, Boot*SE* = 0.009, 95% CI = 0.004, 0.040) accounting for 6.52% of the total effect, and loneliness and mental distress (*β* = 0.099, Boot*SE* = 0.018, 95% CI = 0.065, 0.138) which accounted for 32.25% of the total effect. The total mediating effect was, therefore, 56.35%.

**Table 4:** The indirect effects of social isolation on IS through loneliness and mental distress as mediators (*N* = 1201).

Path models	β	Boot*SE*	BCI 95% CI	Mediated %
Total effect: social isolation → IS	0.307***	0.038	0.232, 0.382	100
Direct effect: social isolation → IS	0.242***	0.039	0.165, 0.318	43.65
Total indirect effect: social isolation → IS	0.065	0.016	0.036, 0.098	56.35
Social isolation → loneliness → IS	0.054	0.016	0.016, 0.096	17.59
Social isolation → mental distress → IS	0.020	0.009	0.004, 0.040	6.52
Social isolation → loneliness → mental distress → IS	0.099	0.018	0.065, 0.138	32.25

Models were adjusted for age, sex, residential status, marital status, level of education, employment status, physical activity, and multimorbidity.

Empirical 95% confidence interval does not overlap with zero.

*β* Unstandardized regression coefficients are reported; *BootSE* bootstrapping standard error; *IS* impaired sleep.

***p˂0.001.

Our secondary analyses (Table 5) showed separate bootstrap-derived 95% CI estimations for the total, direct, and indirect effects of nighttime poor sleep and poor sleep quality. We found that social isolation indirectly affected both nighttime poor sleep and poor sleep quality via significant mediation pathways. Importantly, the total indirect effect of social isolation on poor sleep quality was greater (*β* = 0.098, Boot*SE* = 0.007, 95% CI = 0.008, 0.037, accounting for 74.66% of the total effect) than nighttime poor sleep (*β* = 0.075, Boot*SE* = 0.011, 95% CI = 0.024, 0.067, accounting for 42.94% of the total effect). In another sensitivity analysis (Tables S1, S2), social isolation indirectly affected both IS among those aged 50–64 years and those > 65 years through significant mediation pathways. The total indirect effect of social isolation on IS was greater among 50–64-year-olds (*β* = 0.095, Boot*SE* = 0.026, 95% CI = 0.045, 0.148, accounting for 28.90% of the total effect) than > 65-year-olds (*β* = 0.041, Boot*SE* = 0.018, 95% CI = 0.010, 0.081, accounting for 14.88% of the total effect).

**Table 5:** Indirect effects of social isolation on specific sleep problems through loneliness and mental distress as mediators.

Path models	Nighttime poor sleep (*n* = 1201)				Poor sleep quality (*n* = 1201)			
	β	Boot*SE*	BCI 95% CI	Mediated %	β	Boot*SE*	BCI 95% CI	Mediated %
Total effect: social isolation → IS	0.1756***	0.0253	0.1259, 0.2253	100.00	0.1314***	0.0165	0.0990, 0.1637	100.00
Direct effect: social isolation → IS	0.1002***	0.0262	0.0805, 0.1832	57.06	0.0333***	0.0168	0.0767, 0.1428	25.34
Total indirect effect: social isolation → IS	0.0754	0.0108	0.0242, 0.0670	42.94	0.0981	0.0073	0.0082, 0.0366	74.66
Social isolation → loneliness → IS	0.0159	0.0143	0.0106, 0.0455	9.05	0.0382	0.0096	0.0203, 0.0583	29.07
Social isolation → mental distress → IS	0.0101	0.0050	0.0018, 0.0215	5.75	0.0102	0.0045	0.0018, 0.0198	7.76
Social isolation → loneliness → mental distress → IS	0.0494	0.0121	0.0276, 0.0753	28.13	0.0497	0.0085	0.0344, 0.0676	37.82

Models were adjusted for age, sex, residential status, marital status, level of education, employment status, physical activity, and multimorbidity.

Empirical 95% confidence interval does not overlap with zero.

*β* Unstandardized regression coefficients are reported, *BootSE* bootstrapping standard error, IS impaired sleep.

*p˂0.05, **p˂0.01, ***p˂0.001.

## Discussion

### Principal findings and plausible explanations

This study explored the direct and indirect associations of social isolation with IS through loneliness and mental distress among older adults using multiple serial mediation chain. Regressions found that social isolation independently predicted IS, and the results suggested a dose–response association. The socially isolated were at a higher risk of experiencing suboptimal sleep. This observation is consistent with previously published literature which demonstrated associations between social isolation and sleep disturbances among older adults in Western^14,15^ and Asian societies^45^. Utilizing the China Longitudinal Aging Social Survey among 8456 individuals, Zhang et al.^46^ found that social isolation was related to a higher risk of sleep difficulty. In addition, the World Health Organization (WHO)^47^ has linked social isolation to IS and a lower quantity of sleep among older adults. Socially isolated older adults are more likely to report higher levels of stressful events, anxiety, depressive symptoms, and more chronic diseases than those who are socially attached, which may increase sleep difficulty^46,48^. Social isolation is, therefore, a significant predictor of IS. However, the specific plausible underlying mechanisms linking this meaningful relationship remain unclear.

Our results showed that the social isolation-IS association was mediated via three pathways: loneliness, mental distress, and loneliness and mental distress paths. That is, social isolation was associated with IS by increasing loneliness and mental distress, giving credence to our hypothesis. These path models collectively explained ~ 56% of the association of social isolation with IS. This is the first study to disentangle the complex interrelationships among social isolation, loneliness, mental distress, and IS in old age, particularly in low- and middle-income countries (LMICs). Programmatic approaches to improving sleep health among older adults are essential to public health and social policy efforts^5,16^. Our findings contribute to the extant literature on improving understanding of the mechanistic pathways underlying the association of social isolation with sleep.

This study has demonstrated that loneliness mediated the relationship of social isolation with IS, explaining ~ 18% of the indirect overall effect. This observation fortified our second hypothesis that socially isolated individuals were more likely to report poor sleep via the influence of loneliness. This finding is congruent with previous research suggesting that social isolation was a risk factor for loneliness^49^ and associated with sleep difficulty^47^. Studies have shown a strong correlation between social isolation and loneliness. When older people become socially isolated due, in part, to the greater incidence of living alone or having fewer interactions with family/friends, they tend to feel lonely^50–52^. Lack of social engagement and maintaining healthy social relationships with relevant others may lead to feelings of loneliness, particularly among vulnerable older adults. Feeling extremely lonely can interfere with circadian rhythmic activity and sleep architecture leading to IS^14,53^. For example, among 5698 participants from the English Longitudinal Study of Ageing, Shankar^19^ found that baseline loneliness was associated with an increase in the odds of reporting short sleep and more sleep problems at follow-up. In addition, numerous global studies indicate that daily variations in loneliness are strongly associated with impaired sleep among older people^54,55^. Although this association may be bidirectional, older adults with poor sleep can feel extremely lonely^21^. Social isolation increases the incidence of perceived poor belonging and the rise in feeling lonely, which in turn promotes sleep fragmentation.

The current analysis found mental distress mediating the relationship between social isolation and IS. This finding validates our third hypothesis that social isolation would indirectly relate to IS via mental distress. The mediation effect of this path suggests that mental distress accounted for ~ 7% of the social isolation-IS association. Studies note that many older adults suffer various forms and levels of mental distress when socially isolated from resourceful social relationships and engagements^27,56^. Thus, social isolation potentially leads to poor mental health and dysfunction, resulting in lower energy levels and reduced capacity for an efficient sleep cycle. Previous studies have found that social isolation may affect mood and positively correlate with mental and psychologically-related stressors such as major depressive symptoms, increased anxiety and fatigue levels, and poor concentration^18^. Mental ill-health has been associated with stress and IS, such as difficulty falling asleep, short sleep duration, and daytime exhaustion^57^. A combination of anxiety and stress may reduce the positive effects of social capital on IS^58^.

We showed that social isolation among older adults could affect IS through the serial mediating effects of loneliness and mental distress. This explained ~ 32% of the relationship between social isolation and IS, reinforcing the respective hypothesis. The analysis found that loneliness could positively correlate with mental distress, suggesting that individuals who feel lonely are more likely to experience mental distress. This finding supports previous observations^32,59^. Social isolation adversely affects mental health in many ways. Older adults who are detached from relevant others often experience significant levels of loneliness, which has been underscored as a prime risk factor for poor emotional functioning and psychological distress^12,60^ mainly due to negative perceptions of the social environment, lack of confidence, and poor self-esteem. Increased levels of mental dysfunction are frequently and statistically related to severe sleep difficulties in old age^30,58^. Thus, the growth of loneliness and the associated mental distress sequelae in old age may lead to intense suboptimal quality sleep^14^.

Interestingly, our sleep problem- and age-stratified analyses indicated that the indirect effect of social isolation on IS through loneliness and mental distress was pronounced in poor sleep quality and the 50–64-year-olds compared to nighttime poor sleep and the > 65-year-olds respectively. This is an important finding at least, in part, because, sleep quality is directly related to overall health and quality of life^61^. Poor sleep quality appears to subsume nighttime poor sleep patterns^2,11^ and could seriously be impacted indirectly by social isolation via feeling lonely and experiencing poor mental health. Although sleep problems increase with age^1,62^, the linkages between social isolation and IS through loneliness and mental distress may be heightened among the young old compared with the oldest old given that emotional distress could interfere with the daily economic and social activities which are chiefly undertaken by the young old than the oldest old.

### Public health and policy implications

This study is novel and has significant merits in informing relevant public health and policy decisions to improve sleep health and well-being in old age. Our results extend the relevant literature on the effects of social isolation on IS in older adults in LMICs. Clinicians and family caregivers must pay particular attention to socially isolated and detached older adults who are likely to experience emotional dysfunction. Interventions to embed older adults in resourceful interpersonal relationships may enhance their sense of belonging and potentially improve their experiences of sleep. Practical actions to mitigate loneliness and mental distress alongside specific sleep interventions may be effective options for addressing sleep difficulty in this population. The findings suggest that efforts to reduce social isolation and loneliness among older adults to foster social connections and support may be essential in improving their sleep and well-being.

### Methodological strengths and limitations

The methodological strengths of this study relate to the large sample size and application of serial multiple mediation modeling to disentangle the indirect effect of social isolation on IS, given limited knowledge on this topic in the sub-Saharan African setting. In addition, the construction of a higher-level social isolation measure using multiple distinct variables is notable. The use of probability and multi-stage sampling strategy, with design effect estimation, is an essential methodological strength of this study. Another strength relates to using validated measures with strong psychometric properties, increasing our findings' veracity and confidence. Our study, thus, demonstrates a critical effort to understand the dynamics to address IS in old age.

Despite the strengths, this study has some limitations. First, smoking potentially influences sleep quality, particularly among older adults. Prior research has shown that cigarette smokers are more likely to experience sleep problems than non-smokers^63^. Smoking might, therefore, play a critical confounding role in understanding the association between social isolation and sleep in later life. However, our data set did not capture information on the smoking habits of the respondents, and this constitutes a potential limitation in the current analysis which future studies may consider. Second, our analysis could not infer causal relationships between social isolation and sleep due to the cross-sectional design. Evidence has shown that impaired sleep may lead to more social isolation^33^. Future research should consider longitudinal data to explore the temporal sequence of social isolation and IS. Third, we employed self-reported sleep, social isolation, loneliness, and mental distress data rather than objectively measured outcomes. These may be subject to measurement errors and social desirability bias^20^. Fourth, our participants were limited to six districts from one region. It is, therefore, challenging to ensure the full generalizability of the findings to other older Ghanaian populations. Future studies should include a cross-cultural perspective in exploring the pathways of the link between social isolation and sleep. Finally, residual variables also explain these associations despite adjustment for potential confounders.

## Conclusions

This study involving a nationally representative sample of Ghanaian older adults explored the direct and indirect associations between social isolation and IS. Our data provide empirical evidence that the association of social isolation with impaired sleep is respectively and serially explained by loneliness and mental distress. The study highlights the significance of psychological/emotional factors when exploring the mechanistic pathways for the social isolation-sleep link in later life. Knowledge of the relative impacts of the loneliness and mental distress pathways can be helpful for interventions toward reducing the negative effect of social isolation on IS in old age. Future longitudinal and cross-cultural data are required to affirm these mechanisms and evaluate whether emotional dysfunction could be targeted for social isolation’s effect on IS.

## Supplementary Information

Supplementary Information.

## Data Availability

Those interested in the data and materials in this paper should contact the corresponding authors, Razak M. Gyasi at the email address RGyasi.Research@gmail.com.

## Abbreviations

CHRPE: Committee on Human Research Publication and Ethics
CI: Confidence intervals
IS: Impaired sleep
IPAQ-SF: International Physical Activity Questionnaire short form
LMICs: Low- and middle-income countries
OLS: Ordinary least squares
PA: Physical activity
SSA: Sub-Saharan Africa
UCLA: University of California Los Angeles
WHO: World Health Organization
