# Supplementary material for: A two-mediator serial mediation chain of the association between social isolation and impaired sleep in old age
Source: Sci Rep. 2022 Dec 28;12:22458. doi: 10.1038/s41598-022-26840-5 (PMC9797554; doi:10.1038/s41598-022-26840-5)
Supplement: Supplementary file 1 — Supplementary Information. [file 41598_2022_26840_MOESM1_ESM.docx]

**Supplemental Materials**

| Table S1. Age-stratified regression coefficients in the serial multiple mediation analysis. | | | | | | | | |
| --- | --- | --- | --- | --- | --- | --- | --- | --- |
|  |  |  | 50-64 (*n*=585) | | | ≥65 (*n*=615) | | |
|  | Outcome measure | Predictor variable | β | *t* | 95% CI | β | *t* | 95% CI |
| Loneliness | | Social isolation | 0.206*** | 10.049 | 0.166, 0.246 | 0.127*** | 5.480 | 0.081, 0.172 |
| Mental distress | | Social isolation | 0.015 | 1.308 | -0.008, 0.038 | 0.024* | 2.145 | 0.002, 0.045 |
|  | | Loneliness | 0.578*** | 26.833 | 0.536, 0.620 | 0.578*** | 30.672 | 0.541, 0.615 |
| IS | | Social isolation | 0.234*** | 4.351 | 0.128, 0.339 | 0.235*** | 4.499 | 0.132, 0.337 |
|  | | Loneliness | 0.401*** | 2.679 | 0.695, 0.107 | 0.230 | 1.631 | -0.508, 0.047 |
|  | | Mental distress | 1.323*** | 6.875 | 0.945, 1.701 | 0.724*** | 3.809 | 0.351, 1.098 |
| ***Note:*** IS—impaired sleep  Each model was adjusted for age, sex, residential status, education level, income level, marital status, physical activity, self-rated health, multimorbidity, and functional limitations.  ****p*˂.001; ***p*˂.005; **p*˂.05. | | | | | | | | |

| Table S2. Age-stratified indirect effects of social isolation on IS through loneliness and mental distress as mediators | | | | | | | | | |
| --- | --- | --- | --- | --- | --- | --- | --- | --- | --- |
|  |  | 50-64 (*n*=585) | | | | ≥65 (*n*=615) | | | |
|  | Path models | β | Boot*SE* | BCI 95%CI | Mediated % | β | Boot*SE* | BCI 95%CI | Mediated % |
| Total effect: social isolation → IS | | .3287 | .0519 | .2267, .4307 | 100.00 | .2756 | .0514 | .1746, .3766 | 100.00 |
| Direct effect: social isolation → IS | | .2337 | .0537 | .1282, .3392 | 71.10 | .2346 | .0522 | .1746, .3766 | 85.12 |
| Total indirect effect: social isolation → IS | | .0950 | .0259 | .0454, .1479 | 28.90 | .0410 | .0184 | .0095, .0811 | 14.88 |
| Social isolation → loneliness → IS | | .0826 | .0396 | .0065, .1645 | 25.13 | .0292 | .0202 | -.0729, .0064 | 10.60(ns) |
| Social isolation → mental distress → IS | | .0200 | .0154 | -.0082, .0524 | 6.09 (ns) | .0172 | .0107 | -.0006, .0417 | 6.24(ns) |
| Social isolation → loneliness → mental distress → IS | | .1576 | .0342 | .0968, .2296 | 5.18 | .0530 | .0196 | .0215, .0968 | 19.23 |
| ***Note***: *β*–Unstandardized regression coefficients are reported; BootSE –Bootstrapping standard error; IS–impaired sleep  Models were adjusted for age, sex, residential status, marital status, level of education, employment status, physical activity, and multimorbidity.  Note: Empirical 95% confidence interval does not overlap with zero.  ****p*˂0.001; ***p*˂0.01; **p*˂0.05. | | | | | | | | | |
